# Use of *Metarhizium anisopliae* Chitinase Genes for Genotyping and Virulence Characterization

**DOI:** 10.1155/2013/465213

**Published:** 2013-07-09

**Authors:** Saliou Niassy, Sevgan Subramanian, Sunday Ekesi, Joel L. Bargul, Jandouwe Villinger, Nguya K. Maniania

**Affiliations:** International Centre of Insect Physiology and Ecology (icipe), P.O. Box 30772, Nairobi 00100, Kenya

## Abstract

Virulence is the primary factor used for selection of entomopathogenic fungi (EPF) for development as biopesticides. To understand the genetic mechanisms underlying differences in virulence of fungal isolates on various arthropod pests, we compared the chitinase genes, *chi2* and *chi4*, of 8 isolates of *Metarhizium anisopliae*. The clustering of the isolates showed various groups depending on their virulence. However, the analysis of their chitinase DNA sequences *chi2* and *chi4* did not reveal major divergences. Although their protein translates have been implicated in fungal virulence, the predicted protein structure of *chi2* was identical for all isolates. Despite the critical role of chitin digestion in fungal infection, we conclude that *chi2* and *chi4* genes cannot serve as molecular markers to characterize observed variations in virulence among *M. anisopliae* isolates as previously suggested. Nevertheless, processes controlling the efficient upregulation of chitinase expression might be responsible for different virulence characteristics. Further studies using comparative “*in vitro*” chitin digestion techniques would be more appropriate to compare the quality and the quantity of chitinase production between fungal isolates.

## 1. Introduction

Entomopathogenic fungi (EPF) based products are being developed for the control of insect pests in agricultural systems [1–3]. Entomopathogenic fungi infect their hosts through the cuticle and do not need to be ingested like bacteria, viruses, and protozoa [4]. During the process of infection, EPF secrete chitinase to digest insect cuticle [5–8]. Chitinases are also involved in many other functions of fungal biology, including cellular processes such as conidial germination, hyphal growth, and morphogenesis [9–11]. Chitinase production may be upregulated to solubilise exogenous chitin fibers of both niche competitors (for defence) and nutritional substrates [12–14]. Due to these crucial functions, chitinase genes are suggested as efficient molecular markers for genotyping EPF such as *Metarhizium anisopliae* [15–18].

The entomopathogenic fungus *M. anisopliae* produces at least six types of chitinases [9, 15, 19]. However, the respective role of these proteins in the process of pathogenicity as well as their contribution to virulence on arthropod pests has not been clearly elucidated [20, 21]. Nonetheless, chitinase *chi2* gene isolated from *M. anisopliae* var. *anisopliae* strain E6 has been reported to be responsible for virulence in the genus *M. anisopliae* [22]. Overexpression of *chi2* constructs showed higher efficiency in host killing, while the absence of the same chitinase reduced fungal infection efficiency [20]. Recent studies on differential expression of chitinase genes *in vitro* and *in vivo* established the role of substrate differences in the process of pathogenesis [23]. To understand the role of chitinase genes underlying differences in virulence between fungal isolates, we compared the virulence against various arthropod pests and characterized the chitinase genes of 8 isolates of* M. anisopliae* from the International Centre of Insect Physiology and Ecology (*icipe*)'s Arthropod Germplasm Centre.

## 2. Materials and Methods

### 2.1. Fungal Isolates

Fungal isolates were selected from the *icipe*'s Arthropod Germplasm Centre (Table 1). They were previously bioassayed on 11 arthropod pests belonging to the following taxonomic groups: Diptera, Thysanoptera, Coleoptera, Isoptera, and and Acari (Table 2). Green Muscle, a *Metarhizium anisopliae* var. *acridum* (IMI330189) based biopesticide for the control of locusts (Prior 1997), was included in the study as a reference. Fungal isolates were cultured on Sabouraud Dextrose Agar (SDA) in 9 cm Petri dishes and incubated at 25 ± 2°C in complete darkness for two weeks. Conidia were harvested by scraping the surface using a spatula.

### 2.2. DNA Extraction

For each isolate, 0.1 g of conidia from pure culture was weighed in microcentrifuge tubes on a weighing balance (Mettler AT 261 Delta, Listers 2000). DNA was extracted using a slight modification of the CTAB method described by Doyle and Doyle and resuspended in prewarmed sterile deionized water.

The *chi2* and *chi4* gene fragments were amplified by PCR using published primers (*chi2*: chi2f-GACAAGCACCCGGAGCGC, chi2r-CTTGCTTGACACATTGGTAA; *chi4*: chi4f-GGCTACTGGGAGAACTGGGAC, chi4r-TGTCGCCAARTGTCCARTT) [18, 24]. Primers were purchased from Inqaba Biotec, 525 Walker Street, Muckleneuk, Pretoria (South Africa). Each chitinase gene was amplified separately in 20 *μ*L reactions comprising 1x PCR buffer (Genscript, Piscataway, NJ, USA), 2.5 mM of each dNTP (Genscript), 0.2 picomole of each primer, and 2.5 mM of MgCl_2_, 0.5 units of Taq DNA polymerase (Genscript), and ~25 ng of genomic DNA. PCR amplification was performed in a PTC-100 thermocycler (MJR Inc., Minneapolis, MN, USA) using the following cycling parameters: 30 s initial denaturation at 98°C, followed by 32 cycles of 10 s at 98°C, 20 s annealing, and 90 s at 72°C followed by a final extension of 7 min at 72°C. 

### 2.3. DNA Quantification and Sequencing

The amplification products were separated by electrophoresis in 1% agarose gels containing ethidium bromide (3 *μ*L) in 1 × TAE buffer for 1 h at 70 Vcm^−1^. DNA was visualized under UV light and recorded using a Kodak Gel imaging system (Gel logic 200, Carestream Health, New Haven, CT, USA). The lengths of the amplicon products were estimated by comparison with 1 kb Smart DNA ladder (Noxo, Tallinn, Estonia). The PCR products were purified using QuickClean DNA gel extraction kit (Genscript). Sequences were obtained from Macrogen (Republic of Korea).

### 2.4. Statistical Analysis

Records on the performance of each of the *M. anisopliae* isolates were obtained from *icipe* archives. Virulence data (percentage mortality and lethal time to mortality (LT)) of each isolate was used in the cluster analysis. For each pest, a virulence factor for each isolate was determined by using the average mortality value of the total percentage mortality of all isolates. The same procedure was used for LT values. Data were then subjected to a *k*-mean clustering model to determine the difference in their virulence. The centroid, which is the mean vector of each cluster, was used to define cluster membership of each isolates. The within-groups inertia was used as a criterion to define cluster compactness.

The number of clusters was fixed at 4 (*k* = 4) according to the major taxonomic groups that were considered in this study. Missing values were estimated. A factor analysis based on Spearman correlation (Quartimax rotation) was used to determine the relation between the isolates. The number of iterations performed was 11 and the overall iterations were 200. All statistical analyses were performed using XLSTAT-Pro (Version 7.2, 2003, Addinsoft, Inc., Brooklyn, NY, USA); the significance level was set at *α* = 0.05.

### 2.5. Sequence Diversity and Phylogeny

Chitinase nucleotide sequences were edited and aligned to remove ambiguous base calls before they were translated into proteins using Geneious [25]. A search to identify protein sequences similar to *chi2* and *chi4* was performed using tBLASTx algorithm of NCBI GenBank. Geneious Software was used to estimate phylogeny with the neighbour-joining, minimum evolution, or maximum parsimony method. A dendrogram was constructed using Molecular Evolutionary Genetics Analysis (MEGA) software version 4.0 with 10,000 bootstrap replicates [26]. All methods returned trees with similar topology and approximate bootstrap values; therefore only the neighbor-joining tree is presented. Percentage homology among similar chitinases to *chi2* and *chi4* were computed using MEGA software. 

The 3D structure was predicted using Swiss PdB Viewer, v 4.0.1 (http://www.expasy.org/spdbv/). The conserved residues of the Carbohydrate Insertion Domain (CID) [27] were identified through multiple sequence alignment with the characterized chitinase genes.

## 3. Results

### 3.1. Clustering of *M. anisopliae* Isolates

**Figure 1:**
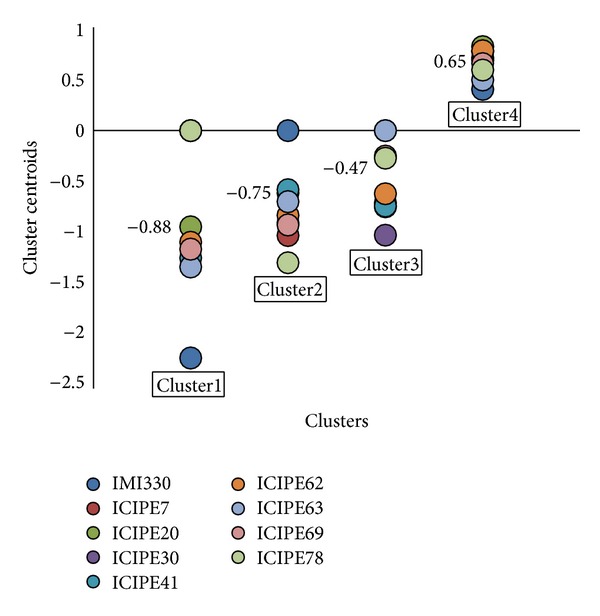
Clustering of *M. anisopliae* isolates based on their virulence (*k* = 4). IMI330189 was added as a reference. The label values represent the average of the centroid for distance comparison.

Considering the taxonomic major groups of host insects: Diptera, Thysanoptera, Acari, and Isoptera, the *k*-mean was fixed at 4, and the analysis of the clusters showed that in cluster1, ICIPE20 (−0.9), ICIPE62 (−1.0), and ICIPE69 (−1.1) were the closest isolates to the centroid (−0.9) as compared to ICIPE7 (0.0), ICIPE30 (0.0), ICIPE78 (0.0), and IMI330189 (−2.2). Cluster2 (average centroid = −0.8) includes ICIPE63 (−0.7), ICIPE20 (−0.9), ICIPE69 (−0.9), and ICIPE30 (−0.6). ICIPE41 (0.6) and ICIPE7 (−0.6) can be suggested in that cluster whereas ICIPE78 (−1.3) and IMI330189 (0.0) were distant to the average centroid. In cluster3, the average centroid is equal to −0.5; ICIPE62 (−0.6) and ICIPE78 (−0.3) have the nearest values, followed by ICIPE69 (−0.2), ICIPE20 (−0.7), ICIPE41 (−0.7), and ICIPE7 (−0.7). ICIPE30 (−1.0), ICIPE63 (0.0), and IMI330189 (0.0) cannot be considered in that cluster as they were distant from the average centroid (−0.5). Finally in cluster4, ICIPE69 (0.7) and ICIPE30 (0.7) have the nearest values, followed by ICIPE7 (0.7) and ICIPE78 (0.6). Although all isolates were agglomerated in one group, ICIPE20 (0.8) and IMI330189 (0.4) are located at the edges (Figure 1).

### 3.2. Clustering of Insects Based on the Virulence Data of the *M. anisopliae* Isolates

The grouping of the arthropods into clusters based on virulence data showed that cluster1 (inertia = 0.0) includes fruit-fly species *C. rosa* and *C. capitata*; cluster2 (inertia = 8.2) comprises ornamental pests such as *F. occidentalis*, *M. sjostedti*, *L. huidobrensis*, and *T. urticae*; cluster 3 (inertia = 9.3) includes five hosts belonging to various taxonomic groups: *C. cosyra*, *P. duboscqi*, *T. evansi*, *M. michaelseni*, and *C. puncticollis*. Cluster 4 with the highest inertia of 73.1% corresponded to the highest diversity of arthropods pests (Table 3).

### 3.3. Relation between the *M. anisopliae* Isolates

Factor analysis using correlation matrix showed various levels of similarity between the isolates based on their performances on the 11 insect pests. ICIPE7 has similarities with ICIPE20, ICIPE69, and ICIPE78 whereas ICIPE20 is closely related to ICIPE41 and ICIPE62; ICIPE 30 is only related to ICIPE7 and ICIPE78, although the correlations were not strong; ICIPE41 was strongly related to ICIPE62, ICIPE63, and ICIPE69; ICIPE62 and ICIPE63 have closed virulence patterns as IMI330189. ICIPE20 and ICIPE41 also are related to IMI330189. There were also similarities in virulence patterns between ICIPE78, ICIPE20, and ICIPE41 (Table 4).

### 3.4. Analysis of Chitinase2 Gene Sequence

**Figure 2:**
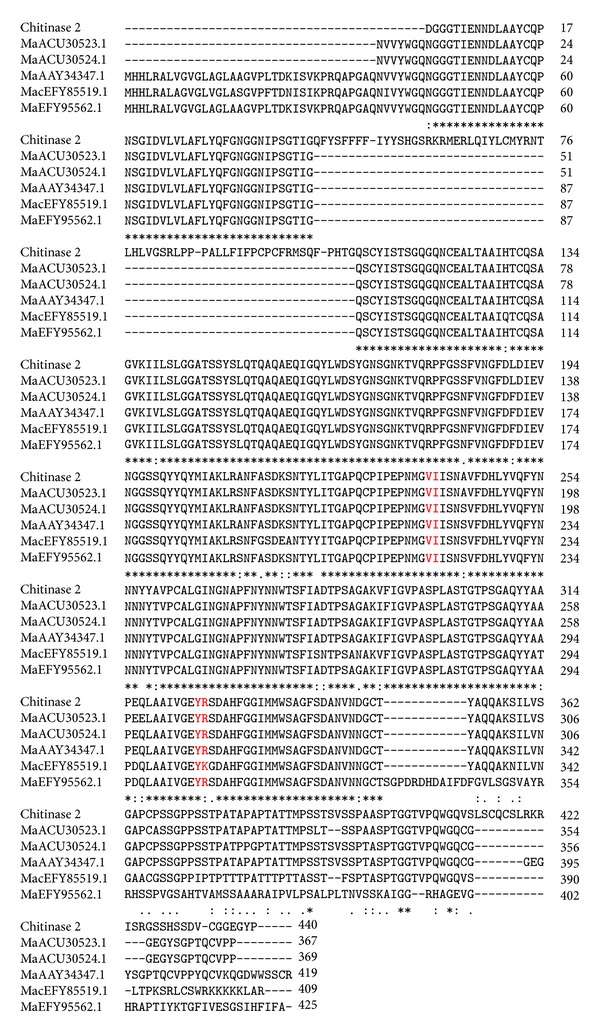
The multiple sequence alignment (Clustal W v2.1) showing the relationship between the Chitinase 2 with similar sequences obtained from the NCBI. The initials represent the species (Ma: *Metarhizium anisopliae*; Mac: *Metarhizium anisopliae* var. *acridium*) followed by their accession numbers as provided in the GenBank. The highlighted residues in red (VI and YR) show the conserved residues of CID.

Comparison of the *chi2* nucleotide sequences from all selected *M. anisopliae* isolates originating from three different parts of Africa showed no differences in the open reading frames composed of 229 amino acid residues. However, when compared with the similar chitinase sequences retrieved from NCBI database, there were differences in amino acid composition (Figure 2).

**Figure 3:**
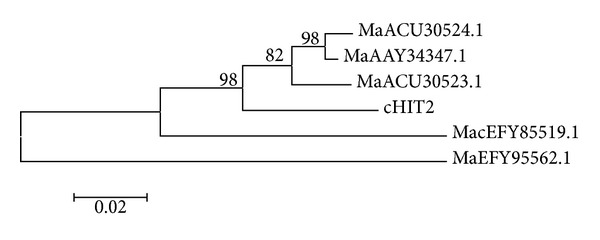
A dendrogram showing the relationships between the *chi2* gene and the related sequences retrieved from the NCBI GenBank.

The phylogenic analysis showed over 95% amino acid identity of chitinase *chi2* sequence. *Metarhizium anisopliae* var. *acridum* strain CQMa 102 (MacEFY85519.1) and *M. robertsii* ARSEF 23 (MaEFY95562.1) were genetically different from other *M. anisopliae* including the *icipe chi2* template from the *icipe* isolates and the other three outgroups M34412 (MaACU30524.1), E6 (MaAAY34347.1), and ARSEF 7524 (MaACU30523) (Figure 3).

### 3.5. Homology Modeling of Chitinase2

**Figure 4:**
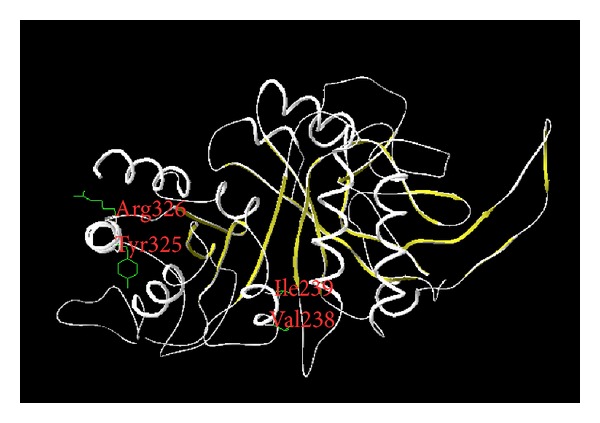
Chitinase2 model as predicted using the Swiss-PdB Viewer. The residues highlighted (Val238 and Ile239; Tyr325 and Arg326) represent conserved residues in the Carbohydrate Insertion Domain (CID) of chitinases.

The Swiss-Pdb Viewer (http://www.expasy.org/spdbv/) server was used to predict the 3D structure of *chi2*. The conserved residues of the Carbohydrate Insertion Domain (CID, Y × R and V × I) were present in all selected *M. anisopliae* isolates that exhibited no differences in their coding regions. In *M. anisopliae* var. *acridum* the “Y × R” motif is replaced by “Y × K” (Figure 4). 

### 3.6. Analysis of Chitinase4 Gene Sequence

**Figure 5:**
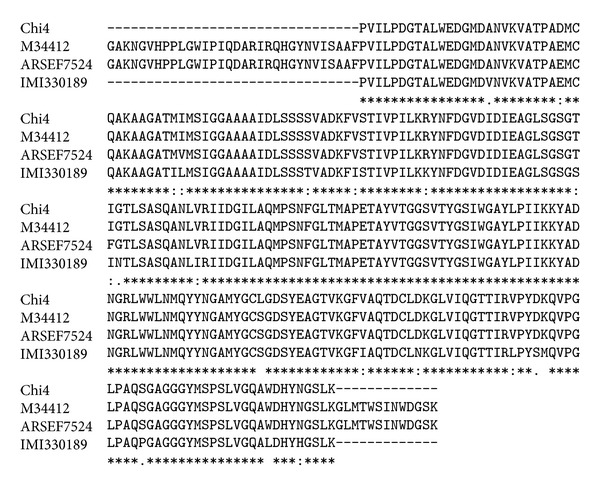
The multiple sequence alignment (Clustal W, v2.1) showing the relationship between the Chitinase4 with similar sequences obtained from the NCBI. The initials represent the species followed by their accession numbers as represented in the GenBank.

All *M. anisopliae* var. *anisopliae* isolates had identical *chi4* nucleotide sequences. After the editing process to remove the ambiguous base calls a BLAST analysis using *chi4* sequence on NCBI GenBank database revealed highest amino acid identities to *M. anisopliae* var. *anisopliae* M34412, ARSEF7524, and *M. anisopliae* var. *acridum* IMI330189 (Figure 5).

## 4. Discussion

The clustering analyses based on virulence data on various taxonomic groups revealed differences between the *icipe*'s isolates. Cluster1 comprises fruit flies *C. rosa* and *C. capitata*, against which ICIPE 20 is most virulent, although other isolates have been reported to be pathogenic [28]. ICIPE20 also fits in Cluster2, which comprises *L. huidobrensis*, *F. occidentalis*, *T. urticae*, and *M. sjostedti* against which it has been reported to be pathogenic [29–32]. Cluster2 also accommodates ICIPE 69 which has been reported to be virulent against thrips [2, 29, 33] and is currently commercialised for the control of insect pests of horticulture in Africa [32]. ICIPE7 which has been reported to be most virulent isolate against *T. urticae* [30] can also be considered in that cluster. Cluster3, on the other hand, includes flies, termites, and mites and therefore involves a larger number of isolates. Previous records on their virulence indicate that ICIPE7, ICIPE20, ICIPE30, ICIPE78, and ICIPE62 could be included in that cluster because of their virulence on *T. urticae*, *M. michaelseni*, and *C. puncticollis* [30, 34, 35]. ICIPE69 has been reported to be the least virulent isolate against *M. michaelseni* [36] and thus cannot be considered in that cluster. This may explain the absence of thrips species in cluster3. Cluster4 comprises 11 arthropod pests, suggesting that each of the isolates is virulent to some extent to each of these pests and their related species. For instance, ICIPE30 has been used for the control of tsetse fly *Glossina* spp. [30, 37]. ICIPE7, which is virulent against mites *T. urticae* and *T. evansi* [30, 38], is also indicated for the control of the tick species *Rhipicephalus appendiculatus* and *R. pulchellus* [39, 40], both belonging to Acari group. ICIPE78, known to be the most virulent isolate for the control of *T. evansi* [37, 41], is closely related to ICIPE7.

Results from the clustering analysis suggest the existence of potential genetic differences in virulence among the isolates. Therefore, molecular investigations on functional genes such as chitinase should be able to illustrate those variations [42, 43]. However in the present study, the comparison of chitinase sequences, *chi2* and *chi4*, among the various *M. anisopliae* isolates did not show differences in nucleotide sequence that could be exploited for genotyping.

All the *M. anisopliae* isolates used in this study showed the same *chi2* and the same *chi4* protein structure despite the fact that they originated from different localities in Africa. Only IMI330189 (*M. anisopliae* var. *acridum*) which originated from Niger had a nonsynonymous substitution in the *chi4* sequence. The analysis of the common predicted structure of the chitinase showed folding patterns and conserved amino acids of the Carbohydrate Insertion Domain (CID) described in many fungal species [9, 27, 44] including NCBI outgroup sequences. 

Chitinase gene *chi2* was reported to be mainly responsible for *M. anisopliae* virulence [20, 23]. The present molecular results suggest either that chitinase genes are differentially regulated (i.e., different expression levels) in different isolates or that there are other parameters that affect the process of infection. Regarding the first hypothesis, *chi2* gene has been reported to be upregulated by chitin (which serves as a carbon source to the fungus) in conditions of fungus autolysis, and is downregulated by glucose [25]. Chitin composition of insect cuticle can affect chitinase production level [23, 45], which would justify the difference in virulence. Since insect pests have special cuticle compositions, the virulence of EPF may vary accordingly, even between life stages [23]. In that regard, Moritz [46] reported that adult thrips and larvae have different cuticle structures, which could explain, in part, the difference in susceptibility to EPF between arthropod pests [32, 47–49]. Posttranscriptional regulation of chitinase genes [50] may also account for the observed virulence difference in our isolates. This needs to be further investigated by comparing chitinase gene expression of isolates with different virulence patterns. Additionally, other relevant factors, such as conidiation and toxin production genes, that affect fungal virulence need to be considered as well. Niassy et al. [32] observed that ICIPE 69 produced more conidia than ICIPE 20 and ICIPE 7 and was virulent to larvae of *F. occidentalis*. Fang et al. [24] demonstrated that gene disruption of a conidiation-associated gene (*cag8*) in *M. anisopliae* resulted in the lack of conidiation on agar plates and on infected insects reduced mycelial growth and decreased virulence, suggesting the involvement of *cag8* in the modulation of conidiation, virulence, and hydrophobin synthesis in *M. anisopliae*. All these gene-regulatory processes need to be considered when developing molecular techniques for genotyping EPF.

## 5. Conclusion

In conclusion, the use of chitinase genes for molecular characterisation of fungal virulence needs to be supported by other markers such as conidiation genes. To understand the difference in virulence between fungal isolates, chitinase gene expression profiling and *in vitro* chitin digestion procedures might be more adequate to compare the quality and quantity of chitinase production. 

## Figures and Tables

**Table 1:** List of *Metarhizium anisopliae* isolates investigated for their chitinase gene; ARSEF 7524, M34412, E6, and ARSEF 324 are out-groups from GenBank.

Species	Isolates	Locality (country)	Origin
*M. anisopliae *isolates from *icipe *	ICIPE 7	Rusinga Island (Kenya)	*Amblyomma variegatum *
	ICIPE 20	Migori-Kenya	Soil
	ICIPE 30	Kendu Bay (Kenya)	*Busseola fusca *
	ICIPE 41	Migori (Kenya)	Soil
	ICIPE 62	Matete (DRC)	Soil
	ICIPE 63	Matete (DRC)	Soil
	ICIPE 69	Matete (DRC)	Soil
	ICIPE 78	Ungoye (Kenya)	*Temnoschoita nigroplagiata *

*M. anisopliae *out-group	ARSEF 7524	Switzerland	Larva, *Agriotes* sp. Coleoptera
	M34412	India	Soil
	E6	Brazil	*Deois flavopicta *
	IMI330189	Niger	*Ornithacris cavroisi *
	ARSEF 324	Queensland, Australia	Acrididae

**Table 2:** List of tested arthropod pests with *M. anisopliae* isolates from *icipe*.

Order	Species	Host plant
Diptera	*Ceratitis rosa* Karsch	Fruit pest
	*Ceratitis capitata *Weidemann	Fruit pest
	*Ceratitis cosyra *Walker	Fruit pest
	*Phlebotomus duboscqi *Neveu-Lemaire	Disease vector in mammals
	*Anopheles gambiae *	Disease vector in mammals
	*Glossina* spp.	Disease vector in mammals
	*Liriomyza huidobrensis* Blanchard	Ornamental pest

Thysanoptera	*Frankliniella occidentalis *Pergande	Ornamental pest
	*Megalurothrips sjostedti *Trybom	Ornamental pest

Coleoptera	*Cylas puncticollis* Boheman	Sweet potato

Isoptera	*Macrotermes michaelseni* Sjostedt	Foraging pest

Acari	*Tetranychus urticae *Koch	Ornamental pest
	*Tetranychus evansi *Baker and Pritchard	Ornamental pest

**Table 3:** Composition of the clusters based on arthropod pests and disease vectors and their susceptibility to the *M. anisopliae* isolates.

Clusters	Cluster1	Cluster2	Cluster3	Cluster4
Within-groups inertia	0.01	8.3	9.7	73.1

Size	2	4	5	11

	*C. rosa *	*F. occidentalis *	*C. cosyra *	*F. occidentalis *
	*C. capitata *	*M. sjostedti *	*P. duboscqi *	*M. sjostedti *
		*L. huidobrensis *	*T. evansi *	*L. huidobrensis *
		*T. urticae *	*M. michaelseni *	*C. rosa *
			*C. puncticollis *	*C. capitata *
				*C. cosyra *
				*P. duboscqi *
				*T. urticae *
				*T. evansi *
				*M. michaelseni *
				*C. puncticollis *

**Table 4:** Spearman correlation matrix between *M. anisopliae* isolates based on their virulence.

	IMI330	ICIPE7	ICIPE20	ICIPE30	ICIPE41	ICIPE62	ICIPE63	ICIPE69	ICIPE78
IMI330		−0.062	**0.499**	0.012	**0.509**	**0.611**	**0.746**	0.298	0.033
ICIPE7			**0.617**	**0.538**	0.401	**0.532**	0.205	**0.662**	**0.726**
ICIPE20				0.393	**0.608**	**0.765**	**0.616**	**0.661**	**0.541**
ICIPE30					0.266	0.349	0.163	0.408	**0.431**
ICIPE41						**0.637**	**0.564**	**0.504**	0.369
ICIPE62							**0.691**	**0.642**	**0.483**
ICIPE63								**0.451**	0.234
ICIPE69									**0.557**
ICIPE78									

In bold, significant values at the level of significance alpha = 0.050 (two-tailed test).
